# Exploring the Extracellular Vesicle MicroRNA Expression Repertoire in Patients with Rheumatoid Arthritis and Ankylosing Spondylitis Treated with TNF Inhibitors

**DOI:** 10.1155/2021/2924935

**Published:** 2021-09-30

**Authors:** Joanna Wielińska, Rachel E. Crossland, Piotr Łacina, Jerzy Świerkot, Bartosz Bugaj, Anne M. Dickinson, Katarzyna Bogunia-Kubik

**Affiliations:** ^1^Laboratory of Clinical Immunogenetics and Pharmacogenetics, Hirszfeld Institute of Immunology and Experimental Therapy, Polish Academy of Sciences, R. Weigla 12, 53-114 Wroclaw, Poland; ^2^Translational and Clinical Research Institute, Faculty of Medical Sciences, Newcastle University, NE1 7RU Newcastle upon Tyne, UK; ^3^Department of Rheumatology and Internal Medicine, Wroclaw Medical University, Borowska 213, 50-556 Wroclaw, Poland

## Abstract

Rheumatoid arthritis (RA) and ankylosing spondylitis (AS) belong to the most common inflammatory rheumatic diseases. MicroRNAs (miRNAs) are small 18–22 RNA molecules that function as posttranscriptional regulators. They are abundantly present within extracellular vesicles (EVs), small intercellular communication vesicles that can be found in bodily fluids and that have key functions in pathological and physiological pathways. Recently, EVs have gained much interest because of their diagnostic and therapeutic potential. Using NanoString profiling technology, the miRNA repertoire of serum EVs was determined and compared in RA and AS patients before and after anti-TNF therapy to assess its potential use as a diagnostic and prognostic biomarker. Furthermore, possible functional effects of those miRNAs that were characterized by the most significant expression changes were evaluated using in silico prediction algorithms. The analysis revealed a unique profile of differentially expressed miRNAs in RA and AS patient serum EVs. We identified 12 miRNAs whose expression profiles enabled differentiation between RA and AS patients before induction of anti-TNF treatment, as well as 4 and 14 miRNAs whose repertoires were significantly changed during the treatment in RA and AS patients, respectively. In conclusion, our findings suggest that extracellular vesicle miRNAs could be used as potential biomarkers associated with RA and AS response to biological treatment.

## 1. Introduction

MicroRNAs (miRNAs, miRs) are a family of single-stranded, noncoding endogenous regulatory RNAs derived from double-stranded precursors, typically composed of 21-23 nucleotides. They are involved in the regulation of gene expression at the posttranscriptional level [1, 2]. They function by the way of complementary binding to the 3′UTR regions of target mRNA via the RNA-induced silencing complex (RISC), resulting in inhibition of the translation process [3]. One miRNA molecule can regulate the expression of several genes (transcripts), and a transcript may have a 3′-UTR region that is recognized by many miRNAs [4, 5]. It is estimated that encoding miRNA genes constitute 1-5% of all the genes in humans and animals [6]. Regulatory miRNAs and exosomes are becoming increasingly important in identification of molecular markers related to pathogenesis and prognosis of disease [7].

Extracellular vesicles (EVs) are lipid membrane-enclosed vesicles, released by cells into the extracellular space. EVs are a heterogeneous collection of exosomes, microvesicles, and apoptotic bodies, ranging in size from 40 nm to 4000 nm. Classification is based on their size, origin, and biological function. EVs are present in blood, saliva, urine, milk, and amniotic fluid and are secreted by all mammalian cell types [8]. Their main function is the transport of lipids, proteins, miRNAs, and mRNAs. Extracellular vesicles are also mediators in intercellular communication and immune-regulatory processes such as bone remodelling, which implicates them in pathogenesis of rheumatic diseases [9]. Although the unique mechanism of immune complex formation remains unclear, it has been observed that synovial exosomes contain citrullinated peptides, which are well-known autoantigens in RA [10]. It has been reported that EVs widely participate in RA development, including antigen presentation and immune complex formation, inflammation, delivery of miRNA, and destruction of extracellular matrix [11]. Moreover, EVs are considered to be promising biomarkers in joint diseases such as RA [12]. Recent studies have indicated a significantly higher level of plasma EVs in RA patients compared to healthy individuals [13]. Other studies established a role of EVs as biomarkers in arthritis by showing an association of exosomal amyloid A and lymphatic vessel endothelial hyaluronic acid receptor-1 with disease activity in RA [14] and identified higher expression of long noncoding RNA, HOX Transcript Antisense RNA (HOTAIR), in serum exosomes of RA cases [15].

Pathogenesis of rheumatoid arthritis (RA) and ankylosing spondylitis (AS) is considered to be multifactorial, with disease susceptibility being associated with genetic, environmental, and stochastic factors. RA and AS are the most common inflammatory rheumatic diseases [16, 17]. They are characterized by different clinical, laboratory, and imaging hallmarks [18, 19]. RA is an autoimmune disease characterized by symmetric and erosive arthritis typically affecting small- and medium-sized joints. It is marked by inflammation of joint synovial tissue, resulting in progression of cartilage and bone tissue damage, ultimately leading to disability [20]. AS is a systemic inflammatory disorder that affects the sacroiliac joints and the spine and can also affect peripheral joints, causing characteristic inflammatory back pain, which can lead to structural and functional impairments [21].

A number of studies have reported that alteration of miRNA profiles may play an important role in the pathogenesis of rheumatic diseases [22, 23], and thus, these profiles may constitute potential biomarkers [24]. Increased expression of miRNAs has been detected in various cell types of RA patients, and miR-146a was shown to mediate negative feedback in the immune response of RA [25]. This specific miRNA was found to be overexpressed in synovial fibroblasts, synovial tissue, synovial fluid monocytes, peripheral blood mononuclear cells, and serum plasma of RA patients [26]. miR-146a regulates gene expression of TNF receptor-associated factor 6 (TRAF6) and IL-1 receptor-associated kinase 1 (IRAK1) in inflammation and participates in a negative feedback loop [27]. Moreover, single-nucleotide polymorphisms (SNPs) in miR-146a may alter its expression. The rs2910164 SNP was found to reduce the expression level of miR-146a, which led to less efficient inhibition of target genes, including two molecules important for RA development, TRAF6 and IRAK1, suggesting that miR-146a rs2910164 could contribute to RA development [28]. Moreover, our previous study in a group of Polish patients with RA showed an association between rs2910164 C variant and higher expression of miRNA-146a in serum after three months of therapy with TNF inhibitors [29].

Despite the fact that anti-TNF treatment constitutes a breakthrough in management of RA and other rheumatic diseases, approximately 30% of patients do not achieve any improvement. Because patients with RA or AS have a variable response to treatment, identification of biomarkers capable of predicting therapeutic response is imperative.

Both RA and AS would benefit from discovery of biomarkers that could be detected when disease is present, distinguish between the two disorders, that are associated with disease progression and outcome, and help to predict the response to treatment.

This study is aimed at investigating whether analysis and comparison of miRNA profiles in patients with RA and AS could be used (i) for detection of diagnostic miRNA markers to distinguish RA from AS and (ii) before and after TNF-*α* inhibitor treatment to predict the outcome and effectiveness of this biological therapy on modulation of proinflammatory response.

## 2. Materials and Methods

### 2.1. Patients

AS patients were classified according to the 1984 modified New York criteria [30]. Adult Caucasians (age ≥ 18 years) included in the study were characterized with high-disease activity (defined as BASDAI ≥ 4 and back pain ≥ 4) before initiation of anti-TNF therapy and failed to respond to at least two nonsteroidal anti-inflammatory drugs (NSAIDs) for at least four weeks at maximum doses (if there were no contraindications). Effectiveness of current drug therapy was assessed using Bath Ankylosing Spondylitis Disease Activity Index (BASDAI), which is based on a 0-10 scale measuring discomfort, pain, and fatigue.

RA patients enrolled in this project were classified according to the 2010 American College of Rheumatology (ACR)/European League Against Rheumatism (EULAR) criteria, as well as the presence of active disease represented by the disease activity score in 28 joints (DAS28 ≥ 5.1). Patients qualified for the study were also before biologic agents therapy initiation and failed to respond to at least two disease-modifying antirheumatic drugs (DMARDs). They were ≥18 years, of Caucasian origin, and with complete medical records.

Exclusion criteria for both RA and AS patients comprised clinically significant impairment of hepatic and renal function, coexistence of connective tissue diseases, infections with hepatotropic viruses, infections resistant to therapy, an ongoing history of cancer or uncontrolled diabetes, alcohol abuse, pregnancy or breastfeeding, and insufficient clinical records.

The ASAS/EULAR criteria were used to assess the clinical outcome of anti-TNF treatment.

All RA patients responded to therapy. Good response was a reduction of the DAS28 score ΔDAS28 > 1.2 to a posttreatment value of DAS28 ≤ 3.2, and moderate response was interpreted as ΔDAS28 > 1.2 and a posttreatment DAS28 > 3.2 or 0.6 < ΔDAS28 ≤ 1.2 and DAS28 ≤ 5.1 [31].

For AS, a good response was determined as a reduction of ΔBASDAI ≥ 2.0 from baseline and BASDAI < 3.0 at the endpoint. A moderate response was defined as a reduction of ΔBASDAI ≥ 2.0 from baseline and BASDAI ≥ 3.0 at the endpoint [32].

The study was approved by the Wrocław Medical University Ethics Committee (identification code KB-625/2016), and written informed consent was obtained from all participants.

Clinical characteristics of the patients are presented in Table 1 as mean and standard deviation (± SD).

### 2.2. Sample Preparation

Serum samples were collected from three patients (two men and one woman, aged: 23-46, mean age: 35) with RA and three patients (two men and one woman, aged: 36-70, mean age: 48) with AS at two-time points before and after three months of anti-TNF treatment initialization. All RA patients were treated with Etanercept. In the AS group, two patients were treated with Etanercept and one with Adalimumab. Further characteristics of patients enrolled in this study are detailed in Table 1. Sera were prepared from whole blood samples, which had been collected in 8.5 ml BD Vacutainer® SST™ II advance blood tubes (Becton, Dickinson and Company, Franklin Lakes, NJ, USA) with clot activator and acrylic gel separation. Samples were centrifuged at 1500 g for 10 min and stored at −70°C.

### 2.3. EV and RNA Isolation

Extracellular vesicles (EVs) were isolated from 3 ml of serum using Total Exosome Isolation Reagent (from Serum) (ThermoFisher Scientific), following supplier's guidelines. EV pellets were stored at -80°C.

The EV pellets were resuspended in particle-free PBS, and RNA was isolated using Total Exosome RNA and Protein Isolation Kit (ThermoFisher Scientific), following supplier's protocol. RNA was concentrated incorporating Amicon Ultra-0.5 centrifugal filter unit with Ultracel-3 membrane (Merck Millipore), according to NanoString recommendations.

RNA concentration was determined using the 2100 Bioanalyzer and the RNA 6000 Pico Kit (Agilent Technologies). While accurate RIN numbers could not be generated, due to the lack of ribosomal RNA in EVs, RNA concentration and peak area were assessed. All RNA was stored at −80°C.

### 2.4. EV Morphology and Size Measurement

To find out whether EVs were properly isolated, EV morphology and size distribution were assessed. For EV morphology, transmission electron microscopy (TEM) was used. 300-mesh grids were filmed with Pioloform® resin (SPI Supplies), carbon coated, and plasma etched before use. EVs were directly applied on the grid, stained with 10 *μ*l of 2% aqueous uranyl, and air-dried. Examination was conducted on a Hitachi HT7800 transmission electron microscope equipped with an Emsis Xarosa camera with Radius software, in cooperation with the Electron Microscopy Research Services, Newcastle University.

For EV size distribution analysis, nanoparticle tracking analysis (NTA) was performed employing a NanoSight LM10 microscope supplied with NTA software version 3.0 (NanoSight Ltd., UK). Background extraction with blur settings and maximum jump distance was applied automatically, and 5 × 60 second recordings were taken for each sample.

### 2.5. miRNA Profiling

Serum EV microRNA expression profiling was performed using the nCounter® Human v3 miRNA Expression Assay kit (NanoString Technologies) as previously described [33]. This code set comprises 98% of microRNA sequences found in miRbase v22 and includes 798 mature microRNAs, six positive and eight negative controls, six ligation controls, and five reference controls. The procedure was performed according to manufacturer's guidelines. Data normalization was performed using nSolver Analysis Software v4.0 (NanoString Technologies), with positive control normalization using the geometric mean and normalization flagging outside the normalization factor range 0.3-3.0. Codeset content normalization was performed using the top 100 microRNAs for normalization, based on geometric mean and flagging outside the normalization factor range 0.1-10.0.

### 2.6. Statistical Analysis

Data normalization and fold change (FC) expression differences between groups were conducted using nSolver v4.0 software (NanoString Technologies). Further analyses were performed using the R software (version 3.6.1) with RStudio 1.2.5001 (RStudio, Inc., USA) applying an analysis pipeline designed by Newcastle University, Haematological Sciences Department. “ggplot2” (v2.1.0) package function was used to construct Volcano plots. Heatmaps with unsupervised clustering were generated using “gplots” (v2.17.0) and “RColorBrewer” (v1.1-2). *p* values between the two groups were calculated using a two-tailed *t*-test. Significance was set at *p* < 0.05.

### 2.7. Target Prediction and Pathway Analysis

To perform gene prediction and pathway analyses for miRNAs obtained from NanoString, we incorporated an approach described by Lou et al. [34]. Potential microRNA gene targets were identified by the miRNet database (http://www.mirnet.ca/) [35]. The STRING database (http://string-db.org) was used to construct networks of protein-protein interactions based on target genes obtained from miRNet [36]. Hub genes, or genes from the protein-protein interaction network that have the highest degree of connectivity, were determined using the Cytoscape software (version 3.7.2) [37]. They were subsequently used as input in the KEGG pathway enrichment analysis performed in the Database for Annotation, Visualization and Integrated Discovery (DAVID, http://david.abcc.ncifc http://rf.gov/) [38].

## 3. Results

### 3.1. NanoString Experimental Setup and MicroRNA Expression Counts

In this study, serum samples from three RA patients and three AS patients before and after administration of biological treatment were analysed. Samples were labelled according to diagnosis (RA or AS), time points of sample collection: before anti-TNF treatment induction (indicated by odd numbers, for example: RA1 and RA3) and after three month of drug administration (indicated by respective even numbers, RA2 and RA4). This labelling method resulted in the following groups: six RA samples, including three before treatment (RA1, RA3, and RA5) and three after therapy (RA2, RA4, and RA6), and similarly six AS samples, three before treatment (AS1, AS3, and AS5) and additional three samples after three months after treatment initialization (AS2, AS4, and AS6).

All NanoString samples passed quality control parameters according to their microRNA expression profiles. A total of 159 microRNAs were positively expressed in >2 RA and AS samples and were included in the final analysis.

### 3.2. MicroRNA Expression Analysis in RA EV Samples before vs. after Therapy

**Figure 1:**
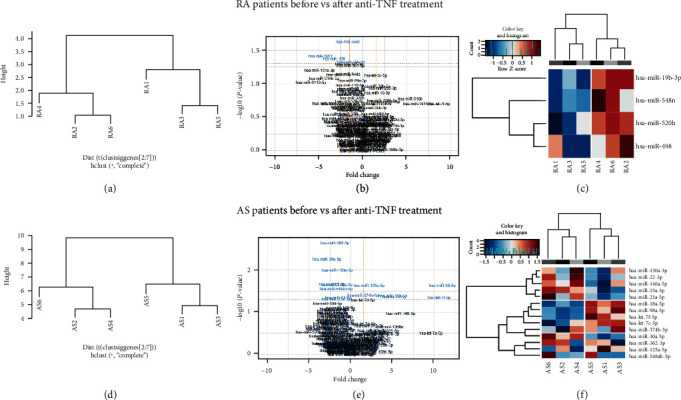
Serum EV microRNA expression in RA and AS patients before vs. after anti-TNF treatment. (a, d) Unsupervised hierarchical clustering analysis. Samples before (1, 3, and 5) vs. after (2, 4, and 6) anti-TNF therapy. (b, e) Volcano plots showing the relationship between fold change and significance for RA and AS patients before and after anti-TNF therapy. The horizontal dashed line indicates cutoff for significance *p* < 0.05 (−log10 *p* value > 1.3) and the vertical lines for fold change ≥ 1.5/≤−1.5. Significantly different miRNAs are highlighted in blue. (c) Heatmaps showing unsupervised hierarchical clustering of differentially expressed miRNAs in serum EVs of patients before (*n* = 3) vs. after Etanercept treatment. The colour scale indicates relative fold change (red: high; blue: low).

Unsupervised hierarchical clustering analysis separated RA serum EV samples before and after three months of anti-TNF treatment as shown in Figure 1(a). EVs from serum collected after three months of anti-TNF therapy were characterized by a unique miRNA signature consisting of four miRNA molecules that were overexpressed (*p* < 0.05), compared to samples collected before treatment: miR-520 h (4.77-fold, *p* = 0.038), miR-498 (3.28-fold, *p* = 0.042), miR-548n (1.66-fold, *p* = 0.023), and miR-19b-3p (1.35-fold, *p* = 0.047) (Figures 1(b) and 1(c), Table 2).

### 3.3. MicroRNA Expression Analysis in AS EV Samples before vs. after Therapy

AS samples were similarly evaluated and unsupervised clustering analysis separated serum EV samples taken before and after three months of anti-TNF treatment (Figure 1(d)). Expression profiling analysis identified 14 miRNAs significantly (*p* < 0.05) differentially expressed in AS after therapy, of which eight were overexpressed: miR-130a-3p, miR-146a-5p, miR-21-5p, miR-22-3p, miR-23a-3p, miR-30a-5p, miR-362-3p, miR-548ah-5p (with FC range 1.49-2.82, *p* value range: 0.002-0.048), while 6 were downregulated: let-7c-5p, let-7f-5p, miR-125a-5p, miR-18a-5p, miR-374b-5p, and miR-98-5p (FC range: 1.48-10.99, *p* value range: 0.023-0.044). The greatest fold change (FC = 10.99) was detected for hsa-miR-98-5p (Figures 1(e) and 1(f), Table 2).

### 3.4. Comparison of RA vs. AS EV miRNA Expression before Anti-TNF Therapy Implementation

**Figure 2:**
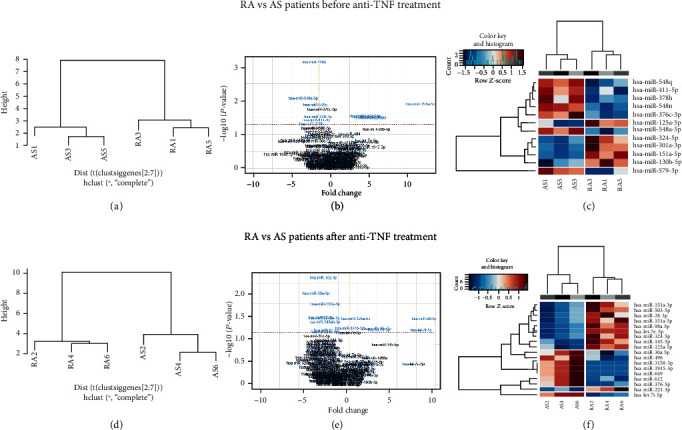
Serum EV microRNA expression in RA vs. AS patients before and three months after anti-TNF treatment. (a, d) Unsupervised hierarchical clustering analysis. RA vs. AS patients before biological treatment initialization and after agent administration. (b, e) Volcano plots showing the relationship between fold change and significance for RA vs. AS patients before and after anti-TNF therapy. The horizontal dashed line indicates cutoff for significance *p* < 0.05 (−log10 *p* value > 1.3) and the vertical lines for fold change ≥ 1.5/≤−1.5. Significantly different miRNAs are highlighted in blue. (c) Heatmaps showing unsupervised hierarchical clustering of differentially expressed miRNAs in serum EVs of RA vs. AS patients before (*n* = 3) and after treatment. The colour scale indicates relative fold change (red: high; blue: low).

To assess whether RA and AS patients can be distinguished based on their serum EV expression profiles, we compared miRNA repertoires at the baseline, before induction of biological treatment (Figure 2(a)). The comparison of RA and AS serum EVs before anti-TNF treatment implementation identified 12 miRNAs with significantly different expression (FC range: 1.08-11.58, *p* value range: 0.001-0.048) (Figures 2(b) and 2(c)). Among those miRNA molecules, higher expression of miR-125a-5p, miR-130b-3p, miR-151a-5p, miR-301a-3p, and miR-324-5p characterized patients with RA (FC range: 4.31-11.58, *p* value range: 0.012-0.031), while miR-376c-3p, miR-378h, miR-411-5p, miR-548a-5p, miR-548n, miR-548q, and miR-579-3p were overexpressed in AS patients, but with a smaller FC range (1.08-3.50, *p* value range: 0.001-0.048) (Figures 2(b) and 2(c), Table 2).

### 3.5. Comparison of RA vs. AS EV miRNA Expression after Therapy

A similar comparison of miRNA repertoires between patients with RA vs. AS was performed on the RA and AS samples collected three months after initialization of anti-TNF treatment. A total of 18 microRNAs were significantly differentially expressed after therapy in RA compared to AS patients (FC range: 1.77-12.76, *p* value range: 0.004-0.050), of which eight were upregulated in AS (miR-7i-5p, miR-1915-3p, miR-30a-5p, miR-3158-3p, miR-379-5p, miR-496, miR-612, and miR-649; FC range: 1.77-3.08, *p* value range: 0.004-0.050) (Figures 2(e) and 2(f), Table 2).

Furthermore, miR-30a was upregulated in AS after therapy in comparison to its expression before treatment and its expression in patients with RA after anti-TNF drug administration. On the other hand, miR-98 was downregulated after initialization of anti-TNF therapy in both cases. Interestingly, miR-151a and miR-125a were characterized with lower expression levels in AS patients compared to those with RA after, as well as before biological therapy.

### 3.6. Identification of Target Genes and Potential Pathways

The potential target genes were analysed separately for upregulated and downregulated microRNAs. 10 hub genes were identified for each of the analysed groups. In RA patients, all upregulated microRNAs after therapy were linked to *TP53*, *EP300*, *PTEN*, *MAPK1*, *STAT3*, *ESR1*, *EZH2*, *CCNB1*, *BRCA1*, and *CASP3*. In AS patients, both upregulated and downregulated microRNAs after treatment were associated with *TP53*, *AKT1*, *MYC*, *UBC*, *EGFR*, and *IL6*. Additionally, downregulated microRNAs after therapy were linked to *UBA52*, *CCND1*, *PTEN*, and *STAT3*, whereas the upregulated ones were associated with *RPS27A*, *MAPK1*, *UBB*, and *VEGFA*. In comparison between RA and AS patients before treatment, downregulated microRNAs were connected to *TP53*, *AKT1*, *MYC*, *UBC*, *EGFR*, *RPS27A*, *MAPK1*, *UBB*, *HSPA8*, and *PTEN*, while upregulated microRNAs were linked to *HSP90AA1*, *CCNB1*, *STAT3*, *CDC5L*, *MDM2*, *CASP3*, *ATM*, *SKP1*, *ACTB*, and *IGF1R*. The hub genes detected in comparison between RA and AS patients after therapy linked to downregulated, as well as upregulated microRNAs were *TP53*, *AKT1*, *MYC*, *EGFR*, and *CTNNB1*, although downregulated microRNAs were also related to *UBC*, *PTEN*, *CCND1*, *HSPA8*, and *VEGFA* and upregulated to *MAPK1*, *JUN*, *HDAC1*, *NOTCH1*, and *MAPK8*.

The KEGG pathway enrichment analysis showed that targets of miRNAs identified as upregulated/downregulated in the treatment-related analyses (RA before vs. after, AS before vs. after) were enriched for pathways associated with response to drugs and cellular response to drugs. Additionally, targets of miRNAs upregulated in AS vs. RA (after therapy) and in AS patients after treatment were enriched in pathways associated with the TNF signalling pathway.

## 4. Discussion

Anti-TNF treatment constitutes a breakthrough in management of rheumatic diseases, although many patients do not achieve significant or any improvement. This makes it essential to find new biomarkers capable of predicting therapeutic response. In the present study, we examined whether serum EV miRNA profiles could be used to distinguish rheumatoid arthritis from ankylosing spondylitis, as well as to predict outcome of TNF inhibitor treatment in both of these rheumatic diseases. We found that in RA patients, four miRNAs (miR-520h, miR-498, miR-548n, and miR-19b-3p) were differentially expressed after anti-TNF treatment, while fourteen were differentially expressed in AS patients (miR-130a-3p, miR-146a-5p, miR-21-5p, miR-22-3p, miR-23a-3p, miR-30a-5p, miR-362-3p, miR-548ah-5p, let-7c-5p, let-7f-5p, miR-125a-5p, miR-18a-5p, miR-374b-5p, and miR-98-5p). Additionally, we identified twelve miRNAs that distinguished the two diseases (miR-125a-5p, miR-130b-3p, miR-151a-5p, miR-301a-3p, miR-324-5p, miR-376c-3p, miR-378h, miR-411-5p, miR-548a-5p, miR-548n, miR-548q, and miR-579-3p).

Recently, there have been many studies investigating EV miRNAs as markers for evaluating RA progression. To date, the association between miRNAs and parameters of disease activity were reported in East Asian populations, specifically in Korean [39] and Chinese [40] RA patients. Wang et al. showed downregulation of exosome-delivered miR-548a-3p in serum of RA patients in contrast with healthy controls, as well as a lower level of miR-548a-3p correlating with higher levels of CRP, RF, and ESR [40]. Similarly, serum exosomal miR-6089 was significantly decreased in RA Chinese patients compared to controls [41]. Regarding plasma exosomes, secretion of miR-17, miR-19b, and miR-121 was significantly higher in RA patients compared to healthy individuals. Moreover, it was found that transport of miR-17 into T cells represses Treg induction and differentiation [42].

MiRNAs delivered by exosomes were also described in the context of their therapeutic potential in RA. miR-150-5p reduced joint destruction by inhibiting angiogenesis mediated by downregulation of matrix metalloproteinase 14 (MMP14) and vascular endothelial growth factor (VEGF) [43]. However, upregulation of exosomal miR-92 boosted bone destruction by blocking apoptosis of fibroblast-like synoviocytes (FLSs) and inflammatory cytokine release [44]. Another microRNA, miR-let-7b, and its ligation to TLR-7 were able to induce joint inflammation through M1 macrophage differentiation [45]. Furthermore, overexpression of miR-221-3p in exosomes isolated from inflamed FLSs might suppress bone formation at erosion sites [46]. These findings provide evidence that exosomal miRNAs can modulate inflammatory responses during RA pathogenesis.

To date, researchers have focused mostly on microRNAs extracted from whole blood, serum/plasma, peripheral blood mononuclear cells (PBMC), and rheumatoid arthritis synovial fibroblasts (RASF) to investigate their role in RA progression [23, 47, 48]. Herein, we present isolated serum exosomes as a novel source of microRNAs. Various techniques can be used to study the miRNA profile. The most common practice is to employ microarray profiling to analyse a wide range of microRNAs on a qRT-PCR instrument [49]. However, a novel technique, NanoString, described previously in a study on serum miRNAs in patients with graft-versus-host disease (GvHD) [33, 50], allows analysis of around 800 microRNAs using digital barcoding technology. This removes the need for reverse transcription or preamplification of RNA, as the technology is able to directly count isolated microRNA molecules. During our study, NanoString was used to assess the repertoire of serum EV microRNAs in RA and AS cases subjected to biological treatment with anti-TNF agents. In the present study, we established for the first time that miR-498, miR-520h, and miR-548n are present in serum EVs of RA patients. Furthermore, expression of miR-7c-5p, miR-7f-5p, miR-362-3p, miR-3746-5p, and miR-548ah-5p has been characterized by us for the first time in AS EVs; in fact, these miRNAs have never been described in any rheumatoid disease at all.

As described in the literature (summary presented in Table 3), miR-155 [51] and miR-146a [52] are among the most widely studied microRNAs in RA and AS patients. Although our analysis did not identify miR-155 expression as significantly different between RA and AS patients, our results regarding miR-146a are consistent with those previously described. Higher expression of miR-146a in RA is well-characterized in multiple components such as synovial fibroblasts [53], CD4+ T cells [54], serum [55], and peripheral blood cells [26]. Moreover, upregulation of miR-146a in various samples from RA patients can distinguish them from osteoarthritis (OA) patients [56]. Interestingly, miR-146a is an NF-*κ*B-dependent gene and controls inflammatory responses through inhibition of IL-1 receptor-associated kinase 1 and TNF receptor-associated factor 6 proteins [25] and downregulation of TLR4/NF-*κ*B pathway [57]. By studying exosomes from rheumatoid arthritis synovial fibroblast cell line, Takamura et al. observed upregulation of miR-146a caused by TNF-*α* stimulation [58]. A positive correlation between the miR-146a level and IL-1*β*, IL-6, and TNF-*α* expression was also observed in AS [59].

There is some evidence to suggest a role for miR-146a in the course of the disease. Filková et al. characterized a decreased level of circulating miR-146a at an early stage of RA, compared to established disease [60]. Furthermore, the expression of miR-146a correlated positively with disease activity in RA [61, 62]. A baseline level of miR-146a was lower in methotrexate responders than in nonresponders [63]. Regarding anti-TNF therapy, previous studies reported increased levels of serum miR-146a in patients under treatment [29, 64]. Besides, Liu et al. demonstrated the potential predictive value of miR-146a measurement for biological agent therapy outcome in RA patients [65].

Recent studies also highlight a role of miR-146a in AS. Significantly higher expression levels have been found in patient serum samples compared to healthy controls [66]. miR-146a overexpression can also cause inhibition of fibroblast proliferation and osteogenic potential while its knockdown blocked disease progression by regulating Dickkopf Wnt Signalling Pathway Inhibitor 1 (DKK1) expression [67]. Other results suggested a positive correlation between miR-146a expression in peripheral blood mononuclear cells and duration of morning stiffness, ESR, CRP, and BASDAI [68]. Prajzlerová et al. also established the role of miR-146a in the pathogenesis of Axial Spondyloarthritis (AxSpA) [69]. Our current study does not show any significant differences in miR-146a expression between RA patients in two time points (before and after three months of the treatment) or RA group compared to AS. However, we detected upregulation of miR-146a-5p in AS cases after anti-TNF therapy. This observation is consistent with previous results obtained by our group for RA patients [29] where the expression level of miR-146a was lower in serum of patients before therapy compared to that after three months of treatment. Taken together, those conclusions implicate miR-146a as a potential noninvasive biomarker in RA and AS prognosis.

Another microRNA, miR-125b, can also serve as a predictor of anti-TNF therapy response [70]. We identified downregulation of miR-125a-5p in AS patient serum EVs after therapy. In contrast, Castro-Villegas et al. observed that patients responding to anti-TNF/DMARD combination therapy exhibited overexpression of miR125b-5p after treatment and lower RF, CRP, TNF*α*, IL-17, and IL-6 levels [64]. Previous studies confirmed that the upregulation of miR-125a and miR-125b directly affects NF-*κ*B [71, 72]. Likewise, decreased miR-125a-5p expression enhanced the protein level of TNFRSF1B and reduced osteoclast activity [73].

On the other hand, Perez-Sanchez et al. found that expression levels of miR-146a-5p, miR125a-5p, miR-151-3p, miR-22-3p, and miR-451a were higher in AS patients compared to psoriatic arthritis patients. Furthermore, miR-146a-5p, miR-125a-5p, and miR-22-3p can distinguish the active and nonactive stage of the disease. Expression of miR-125a-5p, miR-151a-3p, miR-150-5p, and miR-451a was also related to the presence of syndesmophytes in AS patients [74]. Although we demonstrated that miR-146a-5p and miR-22-3p were upregulated in AS patient EVs after therapy, miR-125a-5p was downregulated. Moreover, miR-125a-5p was characterized by lower expression in AS patient EVs compared to RA EVs before and after treatment. Similarly, miR-151-5p was decreased in AS EV samples before and after anti-TNF treatment compared to RA samples. These results implicate miR-125a-5p as a potential diagnostic biomarker in AS.

Little is known about miR-130a and miR-301a-3p. In AS patients after biological therapy, we observed an upregulation of miR-130a. A molecular mechanism involving histone deacetylase 3 (HDAC3) was published by Jiang and Wang [75]. A study on miR-130a in human chondrocytes identified its crucial role in regulating TNF-*α* expression [76]. miR-301a-3p was found to be overexpressed in the PBMCs and associated with Th17 cell frequency in RA patients [77]. We hypothesize that miR-130b-3p and miR301a-3p EV expression may distinguish RA patients from AS patients based on our observation that those microRNAs are higher in RA cases.

To date, publications describing miR-18a referred to osteoarthritis [78, 79], primary Sjögren's syndrome [80], and rheumatoid arthritis [81]. Herein, we demonstrate for the first time downregulation of miR-18a-5p in AS patient EVs after anti-TNF therapy.

We also observed that EVs isolated from sera of RA patients showed overexpression of miR-19b-3p, miR-498, miR-520h, and miR-548n. miR-19b-3p was previously investigated in knee osteoarthritis patients and associated with disease severity [82]. Duan et al. revealed miR-19b-3p involvement in OA through the GRK6-NF-*κ*B pathway [83]. Gantier et al. suggested that miR-19 regulates NF-*κ*B signalling [84]; however, data collected from an experiment with RA FLSs underline the role of miR-19a/b in the stimulation of TLR2 expression [85].

To the best of our knowledge, we showed for the first time that miR-548n and miR-548ah-5p are overexpressed after therapy in RA and AS patients, respectively. Moreover, we identified miR-548n, miR-548ah-5p, and miR-548q as possible biomarkers of AS. Even though no results describing miR-548n, miR-548ah-5p, or miR-548q in rheumatic diseases have been published before, our results are consistent with those reporting about miR-548a-3p, which belongs to the hsa-miR-548 family. miR-548a-3p has previously been shown to be downregulated in serum EVs of RA compared to healthy individuals. Furthermore, expression was negatively correlated with ESR, CRP, and RF levels in patients [40]. In our study, expression of miR-548a-5p was also decreased in RA patients compared to AS patients, before initialization of anti-TNF therapy.

Several studies reported the differential expression of miR-29a and miR-21, making them potentially involved in AS pathogenesis [86–89]. Additionally, overexpression of miR-29a was described in PBMCs from AS patients after etanercept treatment [90]. However, we did not observe differential expression of miR-29a in either RA or AS samples. The analysis showed a significant increase in expression of miR-21 in AS patients after anti-TNF treatment. In contrast, Huang et al. reported greater miR-21 expression in whole blood of patients with AS compared to controls [91]. Recently, Zou et al. showed that upregulation of miR-21 was associated with radiographic severity of AS [92]. The studies regarding RA reported decreased miR-21 level in PBMCs, CD4+ T cells [93], and plasma [94]. Balzano et al. hypothesized that low expression of miR-21-5p was a result of corticosteroids that inhibit NF-*κ*B [94]. Further analyses revealed that miR-21 may be implicated in several signalling pathways such as the IL-34/STAT3/miR-21 pathway, essential for synovial fibroblast survival in RA [95], as well as a mediator between inflammation and bone formation through the JAK2/STAT3 pathway in AS [96]. Additionally, miR-21 plays a role in mediation of RANKL-induced osteoclastogenesis and downregulation of programmed cell death 4 (PDCD4) protein levels [97].

Other microRNA considered to be involved in AS are miR-30a and let-7i. MiR-30a was downregulated in serum/plasma of radiographic axial spondyloarthritis patients compared to healthy individuals [98]. In turn, let-7i was upregulated in T cells [99] and plasma [100] of AS patients compared to controls. In our analysis, both miR-30a-5p and let-7i-5p levels were higher in AS patient EVs after therapy compared to that before treatment and RA cases, respectively. In the study conducted by Lai et al., expression of let-7i in T cells was also positively correlated with the Bath Ankylosing Spondylitis Radiology Index (BASRI) of the lumbar spine, a scoring system which we did not include in our analysis. The authors concluded that overexpression of these microRNAs suppressed TLR-4 expression, which led to downregulation of TLR-4 [99].

The present work is in line with the previous finding identifying miR-23a as a potential predictor of etanercept response [101]. miR-23b can repress IL-17-associated autoimmune inflammation in human fibroblast-like synoviocytes [102]. This observation was confirmed for miR-23a in articular cartilage tissues from RA patients [103].

In summary, to the best of our knowledge, the present study identified for the first time 12 microRNAs differently expressed in serum EVs between RA and AS patients before biological agent administration. miR-125a-5p, miR-130b-3p, miR-151a-5p, miR-301a-3p, and miR-324-5p were upregulated in RA EVs, and miR-376c-3p, miR-378h, miR-411-5p, miR-548a-5p, miR-548n, miR-548q, and miR-579-3p in AS EVs. We believe that these microRNAs have the potential to distinguish RA pathogenesis from AS. The pathway prediction analysis performed using mirPATH v.3 DIANA tools (data not shown) found that all the microRNAs are involved in the Wnt signalling pathway.

## 5. Conclusions

Our analysis has revealed a unique profile of differentially expressed miRNAs in RA and AS patient serum EVs, both before and three months after anti-TNF treatment administration. These results suggest that EV miRNA profiling of RA and AS patients can be used for detection of diagnostic and predictive biomarkers. They confirm a potential role of these miRNAs in distinguishing the two diseases and in the prediction of response to treatment in RA and AS patients. Nevertheless, the results reported herein should be considered as a pilot study that was conducted on a limited number of patients; therefore, validation in larger verification cohorts is required.

## Figures and Tables

**Table 1:** Characteristics of RA and AS patients included in the study.

	RA (*N* = 3)	AS (*N* = 3)
Age [years]	34.67 ± 11.50	5
Sex (female/male)	1/2	1/2
Disease duration [years]	9.333 ± 9.074	15.33 ± 15.63
BMI	27.02 ± 2.728	26.47 ± 8.474
VAS baseline [mm]	82.67 ± 6.429	73.00 ± 18.38
VAS after therapy [mm]	30.00 ± 20.00	19.67 ± 11.68
DAS28 baseline	5.61 ± 0.71	—
DAS28 after therapy	1.85 ± 1.20	—
BASDAI baseline	—	7.90 ± 0.36
BASDAI after therapy	—	2.00 ± 0.87

BMI: body mass index; VAS: visual analogue scale; DAS28: disease activity score 28, BASDAI: Bath Ankylosing Spondylitis Disease Activity Index; RA: rheumatoid arthritis; AS: ankylosing spondylitis.

**Table 2:** Comparison of differentially expressed miRNAs in RA and AS patients before and after three months of the anti-TNF treatment. Statistical characteristics of depicted miRNAs. FC: fold change.

RA samples before vs. after therapy			AS samples before vs. after therapy			RA vs. AS patients before anti-TNF treatment			RA vs. AS patients after anti-TNF treatment		
miRNA	*p*	FC	miRNA	*p*	FC	miRNA	*p*	FC	miRNA	*p*	FC
hsa-miR-19b-3p	0.047	-1.35	hsa-let-7c-5p	0.042	5.21	hsa-miR-125a-5p	0.028	4.31	hsa-let-7e-5p	0.005	6.13
hsa-miR-548n	0.023	-1.66	hsa-let-7f-5p	0.044	10.61	hsa-miR-130b-3p	0.031	4.91	hsa-let-7i-5p	0.004	-1.77
hsa-miR-520 h	0.038	-4.77	hsa-miR-125a-5p	0.023	2.23	hsa-miR-151a-5p	0.012	11.58	hsa-miR-125a-5p	0.020	6.00
hsa-miR-498	0.042	-3.28	hsa-miR-130a-3p	0.010	-1.65	hsa-miR-301a-3p	0.029	4.61	hsa-miR-145-5p	0.012	12.76
			hsa-miR-146a-5p	0.022	-1.67	hsa-miR-324-5p	0.031	5.45	hsa-miR-151a-3p	0.027	3.42
			hsa-miR-18a-5p	0.040	5.05	hsa-miR-376c-3p	0.018	-1.08	hsa-miR-151a-5p	0.027	2.96
			hsa-miR-21-5p	0.045	-1.49	hsa-miR-378 h	0.048	-2.47	hsa-miR-1915-3p	0.033	-2.62
			hsa-miR-22-3p	0.022	-2.22	hsa-miR-411-5p	0.037	-1.77	hsa-miR-221-3p	0.021	1.62
			hsa-miR-23a-3p	0.048	-1.74	hsa-miR-548a-5p	0.008	-3.50	hsa-miR-28-3p	0.044	3.04
			hsa-miR-30a-5p	0.005	-2.82	hsa-miR-548n	0.001	-2.11	hsa-miR-30a-5p	0.050	-2.27
			hsa-miR-362-3p	0.002	-1.86	hsa-miR-548q	0.013	-1.95	hsa-miR-3158-3p	0.033	-2.62
			hsa-miR-374b-5p	0.040	1.48	hsa-miR-579-3p	0.029	-1.60	hsa-miR-324-5p	0.019	4.56
			hsa-miR-548a-5p	0.028	-1.72				hsa-miR-379-5p	0.039	-2.84
			hsa-miR-98-5p	0.023	10.99				hsa-miR-496	0.030	-2.83
									hsa-miR-503-5p	0.012	2.90
									hsa-miR-612	0.024	-3.08
									hsa-miR-649	0.033	-2.62
									hsa-miR-98-5p	0.006	7.32

**Table 3:** MicroRNAs differently expressed in RA and AS patients compared to healthy controls.

miRNA	Disease	Country	Ethnicity	Source	No. of cases/controls	Investigation method	Changes of miR expression	Target gene	References
miR-146a	RA	Egypt	Arab	Whole blood	25/25	qPCR	↑	—	[104]
	RA	Japan	Asian	PBMC	6/5	qPCR^∗^	↑	—	[62]
	RA	China	Asian	PBMC	69/69	qPCR	↑	—	[105]
	RA	Egypt	Arab	PBMC	52/56	qPCR	↑	—	[55]
	RA	Egypt	Arab	PBMC	70/60	qPCR	↑	—	[106]
	RA	Canada	—	PBMC	11/10	qPCR	↑	—	[107]
	RA	USA	—	PBMC	16/9	qPCR	↑	*TRAF6*, *IRAK1*	[26]
	RA	Switzerland	European	Serum	34/16	qPCR	↓	—	[60]
	RA	Poland	European	Serum	13/16	qPCR	↓	—	[29]
	RA	USA	European	Plasma	168/91	qPCR	↑	—	[108]
	RA	China	Asian	Plasma	25/20	qPCR^∗^	↓	—	[109]
	RA	Japan	Asian	Plasma, synovial fluid	30/30	qPCR	↑	—	[61]
	RA	China	Asian	Synovial tissue	17/3	qPCR^∗^	↑	—	[102]
	RA	China	Asian	FLS	12/10	qPCR	↓	*TLR4*	[57]
	RA	Japan	Asian	CD4+ T	33/12	qPCR	↑	*FAF1*	[54]
	RA	Germany	European	Treg	61/49	qPCR	↓	*STAT1*	[110]
	AS	China	Asian	Serum	70/68	qPCR^†^	↑	—	[66]
	AS	Spain	European	Plasma	53/57	qPCR^‡^	↑	—	[74]
	AS	Switzerland	European	Plasma	24/29	qPCR^∗^	↓	—	[69]
	AS	China	Asian	Hip capsule	30/30	qPCR	↑	*DKK-1*	[67]
miR-155	RA	Canada	Native American	Whole blood	18/12	qPCR	↑	—	[111]
	RA	Japan	Asian	PBMC	6/5	qPCR^∗^	↑	—	[62]
	RA	China	Asian	PBMC	45/25	qPCR	↑	*SOCS1*, *TNFa*, *IL-1B*	[112]
	RA	Canada	—	PBMC	11/10	qPCR	↑	—	[107]
	RA	USA	—	PBMC	16/9	qPCR	↑	—	[26]
	RA	China	Asian	PBMC, FLS	26/23	qPCR^∗^	↑	*IKBKE*	[113]
	RA	Egypt	Arab	Serum	100/100	qPCR	↑	—	[114]
	RA	Japan	Asian	Plasma, synovial fluid	30/30	qPCR	↑	—	[61]
	RA	China	Asian	Plasma	25/20	qPCR^∗^	↓	—	[109]
	RA	USA	European	Plasma	168/91	qPCR	↑	—	[108]
	RA	China	Asian	FLS	89/49	qPCR	↑	*FOXO3a*	[115]
	RA	UK	European	CD14+ T	9/8	qPCR^∗^	↑	—	[116]
	RA	Germany	European	Treg	61/49	qPCR	↓	—	[110]
	RA	UK	European	Monocyte	24/22	qPCR	↑	*CCR2*, *CCR7*	[117]
	AS	China	Asian	Serum	70/68	qPCR	↑	—	[66]
miR-21	RA	China	Asian	Plasma	25/20	qPCR^∗^	↑	—	[109]
	RA	Italy	European	Plasma	28/20	qPCR	↓	—	[94]
	RA	China	Asian	CD4+ T, PBMC	25/20	qPCR	↓	*STAT3*, *STAT5*	[93]
	AS	China	Asian	Whole blood	122/122	qPCR	↑	*PDCD4*	[91]
miR-22	RA	China	Asian	RASF	40/40	qPCR	↓	*SIRT1*	[118]
	RA	UK	European	Serum	12/12	Microarray	↑	—	[119]
	RA	China	Asian	FLS	48/30 (OA)	qPCR	↓	*Cyr61*	[120]
	AS	Spain	European	Plasma	53/57	qPCR^‡^	↑	—	[74]
miR-30a	RA	China	Asian	Synovial tissue	7/12 (OA)	qPCR	↓	*Beclin-1*	[121]
	AS	USA	—	Plasma	15/5	Microarray	↓	—	[98]
miR-125a	RA	China	Asian	Plasma	25/20	qPCR^∗^	↓	—	[109]
	RA	Japan	Asian	Plasma	102/104	qPCR^∗^	↑	—	[122]
	RA	USA	European	Plasma	168/91	qPCR	↑	—	[108]
	AS	Spain	European	Plasma	53/57	qPCR^‡^	↑	—	[74]
miR-221	RA	Egypt	Arab	PBMC	30/20	qPCR	↑	—	[123]
	RA	China	Asian	Serum, FLS	22/18	qPCR	↑	—	[124]
	AS	Switzerland	European	Plasma	24/29	qPCR^∗^	↓	—	[69]
miR-29a	RA	China	Asian	Serum, synovial tissue, FLS	20/10	qPCR	↓	*STAT3*	[87]
	AS	Switzerland	European	Plasma	24/29	qPCR^∗^	↓	—	[69]

↑/↓: up/downregulation; RA: rheumatoid arthritis; AS: ankylosing spondylitis; PBMC: peripheral blood mononuclear cell; FLS: fibroblast-like synoviocytes; RASF: RA synovial fibroblasts; ^∗^microarray screening; ^†^sequencing screening; ^‡^NanoString screening.

## Data Availability

All relevant data are within the manuscript. More detailed information cannot be shared publicly because it allows identification of the patients. It can be accessed from the Hirszfeld Institute of Immunology and Experimental Therapy (contact via katarzyna.bogunia-kubik@hirszfeld.pl) for researchers who meet the criteria for access to confidential data.
